# CD4^+^CD25^High^ Treg cells in HIV/HTLV Co-infected patients with neuropathy: high expression of Alpha4 integrin and lower expression of Foxp3 transcription factor

**DOI:** 10.1186/s12865-015-0116-x

**Published:** 2015-09-02

**Authors:** Raquel Matavele Chissumba, Suse Dayse Silva-Barbosa, Ângelo Augusto, Cremildo Maueia, Nédio Mabunda, Eduardo Samo Gudo, Nilesh Bhatt, Ilesh Jani, Wilson Savino

**Affiliations:** National Institute of Health, Ministry of Health, Av. Eduardo Mondlane 1008, 2nd floor, Maputo, Mozambique; Laboratory on Thymus Research, Oswaldo Cruz Institute, Oswaldo Cruz Foundation, Rio de Janeiro, Brazil

## Abstract

**Background:**

Regulatory CD4 T cells (Tregs) are critical in maintaining the homeostasis of the immune system. Quantitative or phenotypic alterations and functional impairment of Tregs have been associated with the development of pathologies including those of the central nervous system. Individuals with HIV-1/HTLV-1 co-infection are more prone to develop neurological complications. The aim of this study was to characterize phenotypically Treg cells in HIV-1/HTLV-1 co-infected Mozambican individuals presenting neurological symptoms.

**Methods:**

A cross-sectional study was conducted among HIV-infected individuals presentingneurological symptoms, with and without HTLV co-infection, and blood donors. Peripheral bloodmononuclear cells were stained with monoclonal antibodies conjugated with fluorochromes to quantifyTregs and activated T cells by four colors flow cytometry.

**Results:**

Higher Treg cell frequency (10.6 %) was noted in HIV-1/HTLV-1 co-infected group with neurological symptoms when compared to HIV-1 mono-infected group with neurological symptoms (0.38 %, *p* = 0.003) and control group (0.9 %, *p* = 0.0105). An inverse correlation between Foxp3 and CD49d expression was observed in all study groups (rh = −0.71, *p* = 0.001). In addition, increased levels of Treg cells in co-infected patients were strongly associated with total activated CD4 T cells (rh = 0.8, *p* = 0.01).

**Conclusion:**

Treg cells in co-infected patients present phenotypic alterations and might have dysfunction marked by low expression of Foxp3 and increased expression of molecules not frequently seen on Treg cells, such as CD49d. These alterations may be related to (1) changes in Treg cell trafficking and migration, possibly making those cells susceptible to HIV infection, and (2) inability to control the activation and proliferation of effector T lymphocytes.

## Background

The Human Immunodeficiency Virus type 1 (HIV-1) is the retrovirus that causes a most severe secondary immunodeficiency, the Acquired Immunodeficiency Syndrome (AIDS). Co-infections with other retroviruses are common, particularly those following the same route of transmission, as the Human T-lymphotropic Virus type 1 (HTLV-1). A recent study conducted in Maputo city, Mozambique, found a prevalence of HIV-1/HTLV-1 co-infection of 4.5 % among antiretroviral therapy (ART) naïve HIV-positive patients [1].

Since both HIV-1 and HTLV-1 have tropism for CD4 T cells, it is relevant to understand how the presence of HTLV-1 can modulate the pathophysiology of HIV-1 infection. Individuals with HIV-1/HTLV-1 co-infection are at a higher risk for developing diseases associated with both viruses. These patients are more prone to develop neurological complications, particularly HTLV-1 associated myelopathy/ tropical spastic paraparesis (HAM/TSP) [2, 3] and may progress faster to AIDS with higher levels of activation markers, despite their higher CD4 T cell counts [4].

Chronic immune activation is a feature of HIV pathogenesis. CD4^+^ T regulatory (Treg) cells are critical to control systemic immune activation. Their suppressive effect is associated with the density of expression of the transcription factor *forkhead box P3* (Foxp3). Moreover, recent findings suggest that a higher suppressive response is achieved when CD4^+^CD25^High^ cells are depleted with the anti-CD49d monoclonal antibody, which recognizes the α4 integrin chain of VLA-4 (α4β1) and LPAM-1 (α4β7) [5].

In the context of HIV infection, there is no consensus regarding the role of Tregs. It seems that Tregs in chronic infection exert simultaneous and paradoxal regulatory effects [6]. Lower frequencies of Tregs have been reported as being associated with higher levels of T cell activation [7, 8]. Conversely, it has been shown that Tregs may prevent protective anti-HIV immune response favoring chronicity [6, 9]. In other contexts, functional or quantitative alterations of Treg cells have been associated with development pathologies, including those of the central nervous system (CNS). Actually, several authors have suggested that the neuroinflammatory alterations observed in HTLV mono-infected patients with HAM/TSP, affecting mainly the brain and the spinal cord [10], are associated with Treg cell dysfunction [11, 12].

Although there are published data characterizing Treg cells in mono-infection by HIV-1 or HTLV-1, so far no data are available regarding the impact of HTLV-1 infection on the regulation of the immune response to HIV-1, particularly in patients with neurological symptoms. Our hypothesis was that in presence of co-infection with HTLV-1, HIV-1 infected patients progress with increased immune dysfunctions, including that of Treg cells, as compared with HIV mono-infected patients. Herein we aimed to phenotypically characterize Treg cells in HIV-1/HTLV-1 co-infected individuals presenting neurological symptoms.

## Methods

### Study population

Sixteen HIV-infected patients attending an HIV outpatient clinic in Centro de Saúde do Alto Maé, a primary health care center in Maputo City, Mozambique were enrolled in this study, from November 2009 to February 2010. Among these patients, eight were co-infected with HTLV-1/2. These patients presented one or more of the following neurological symptoms: weakness of the lower limbs, chronic spastic paraparesis, peripheral neuropathy, sensorial symptoms, hyperreflexia of the lower and upper limbs, cranial neuropathy, reduced libido, bladder disturbance and cognitive failures. The HIV clinical stage was defined according to WHO guidelines for African region [13]. Additionally, five healthy individuals were recruited as a control group for the study at the blood bank service at the Maputo Central Hospital.

The Mozambican National Health Bioethics Committee (CNBS) approved the study. Written consent to publish was obtained from the study participants. Pregnant women were not included in the study. The diagnosis of neurological symptoms was performed by a specialist clinician who routinely followed-up HIV infected patients. All clinical diagnosis was double checked by a second clinician.

### Specimen collection and preparation

For each volunteer 10 ml of venous blood was obtained using a vacutainer EDTA tube (Vacutainner, Becton Dickinson, San José, USA). A volume of 100 μl was used for immunophenotyping of CD4 T, CD8 T, B and NK cells. The remaining was centrifuged to separate plasma and peripheral blood mononuclear cells (PBMC) by using the density gradient Ficoll Hypaque (Life Sciences, Uppsala, Sweden). The number of viable cells was determined by the exclusion method using Trypan Blue.

### HIV-1 and HTLV-1 diagnosis

HIV-1/2 diagnosis was performed using the Mozambican national algorithm for HIV testing, which consists of two sequential rapid immunochromatographic tests for detecting anti-HIV-1/2 antibodies. First, screening was performed using the *Determine HIV*-*1*/*2* (Abbott Laboratories, Japan). Non-reactive specimens were classified as negative. Reactive specimens in the screening assay were confirmed by a second test, *Uni*-*Gold HIV* (Trinity Biotech, Ireland).

HTLV-1/2 screening was performed using a sandwich Enzyme-Linked ImmunoSorbent Assay (ELISA; MP Diagnostic HTLV-I/II ELISA 4.0 - MP Biomedicals Asia Pacific Ltd). Non-reactive specimens were classified as negative, while reactive samples were confirmed by an in-house nested RT-PCR targeting the *pX* gene.

### Flow cytometry

Immunophenotyping of CD4 T, CD8 T, B and NK cells was performed on whole blood samples using the MultiTest antibodies and TruCount tubes (Beckton Dickinson, San Jose, CA, USA). Absolute and relative counts of these lymphocyte subpopulations were obtained following a lyse-no-wash protocol with Trucount beads [14]. Briefly, 20 μL of each panel of antibodies conjugated with different fluorochromes, CD3-FITC/CD8-PE/CD45-PerCP/CD4-APC or CD3-FITC/CD16 + CD56-PE/CD45-PerCP/CD19-APC (both from BD Biosciences) were added to 50 μl of whole blood samples in TruCount tubes. After 15 min, the BD FACS lysing solution was added to lyse red blood cells. After additional 15 min of incubation, samples were ready to be acquired using the FACSCalibur^™^ flow cytometer and MULTISET^™^ software (both from Beckton Dickinson). A total of 15,000 events were acquired for each sample. Analysis was performed using the MULTISET^™^ software.

The remaining immunophenotyping was performed using PBMC stained with fluorochrome conjugated monoclonal antibodies (all from Beckton Dickinson) USA, using the cocktail of antibodies with the following specificities and associated fluorochromes: CD4-PerCP, CD8-PerCP, CD25-FITC, CD49d-APC, HLA-DR-APC, and Foxp3-PE. Mouse immunoglobulin isotypes conjugated with PerCP, PE, FITC or APC were always used as negative control for non-specific binding. For each patient, approximately 1 × 10^6^ cells were firstly treated with 5 μL of human AB serum (Sigma-Aldrich, St. Louis, USA) to avoid non-specific binding. After incubation, a final volume of 20 μL of mixed antibodies in PBS was added to each sample. Cells were then washed in a staining buffer (PBS with fetal calf serum 10 %), and fixed with 300 μL of PBS solution containing paraformaldehyde 1 %. Unrelated Ig isotype fluorochrome-matched antibodies were used as negative controls.

For the measurement of Foxp3, cells were fixed, permeabilized, blocked and treated with 20 μL of anti-human Foxp3 monoclonal antibodies (clone PCH101, eBioscience) following the manufacturer’s instructions. Samples were incubated, washed and re-suspended in staining buffer for analysis. A total of 2 × 10^5^ events were collected in the lymphocyte region gated based on cell granularity and size. The data were acquired using CellQuest^™^ software (Beckton Dickinson) and analyzed with *Summit*, version 4.3 (Dako Corporation, Colorado, USA).

**Fig. 1:**
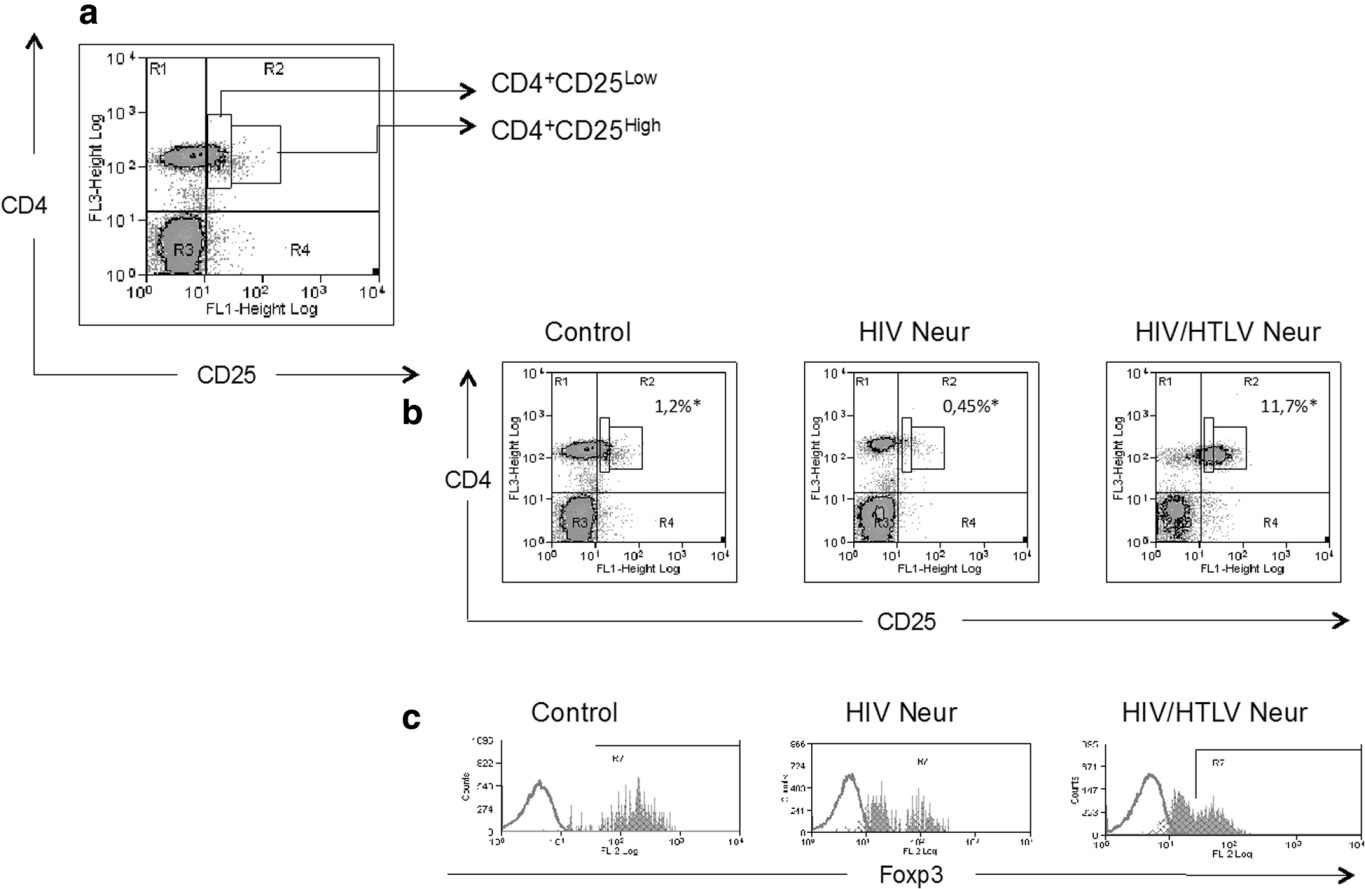
Expression of CD25 in CD4^+^ lymphocytes and representative analysis of FoxP3. In (**a**) dot plot representing the regions, CD4^+^CD25^Low^ and CD4^+^CD25^High^. In (**b**) graphics representing the same regions in “a” in the different study groups control, HIV Neur and HIV/HTLV Neur. For each group we show the median of CD4^+^CD25^High^ cells, in relation to the total number of lymphocytes. In (**c**) histograms representatives of FoxP3 expression within the CD4^+^CD25^High^ region in the study groups. Control (*n* = 5): healthy individuals; HIV Neur (*n* = 8): HIV mono-infected group presenting neuropathy; HIV/HTLV Neur (*n* = 8): group co-infected by HIV-1 and HTLV-1 presenting neuropathy

For Treg identification, two distinct regions of CD4^+^ T cells, expressing different levels of the CD25 marker where separated, (1) CD4^+^CD25^Low^ and (2) CD4^+^CD25^High^. Isotype controls were used to set these limits. Treg cells were identified as FoxP3 positive cells within the CD4^+^CD25^High^ gate (Fig. 1).

### Statistical analysis

Statistical analysis was performed using *STATA 10.1* (Statacorp, College Station, TX, USA) and GraphPad Prism (version 4.0b; GraphPad Software, San Diego, CA). The *Kruskal*-*Wallis* test was used to compare medians among different groups. The analysis of correlations between two variables was performed by the *Spearman Rank correlation*. Difference or correlation with *p*-values less than 0.05 were considered statistically significant.

## Results

### Clinical and demographic characteristics

Table 1 summarizes clinical and demographic features of study participants. There was a predominance of males in controls (5/5) and HIV-infected with neurological symptoms (HIV Neur) group (5/8), when compared to HIV/HTLV co-infected with neurological symptoms (HIV/HTLV Neur) group (2/8). All patients in the group HIV Neur and six in HIV/HTLV Neur group received stavudine (d4T) as part of their first line ART. Four patients in the HIV Neur group and seven in HIV/HTLV Neur group were in stage I of HIV disease; two patients in HIV Neur group and one in HIV/HTLV Neur were in stage II of HIV disease; and two patients in the HIV Neur group were in the stage IV of HIV disease.

**Table 1:** Clinical and demographic characteristics of the study population

Group	Age (Years)	Gender	BMI (kg/m^2^)	WHO stage	Viral load (copies/ml)	d4T as part of initial ART regimen	Duration of ART (months)
Control	N-A	M	N-A	N/A	N/A	N/A	N/A
Control	N-A	M	N-A	N/A	N/A	N/A	N/A
Control	N-A	M	N-A	N/A	N/A	N/A	N/A
Control	N-A	M	N-A	N/A	N/A	N/A	N/A
Control	N-A	M	N-A	N/A	N/A	N/A	N/A
HIV Neur	57	F	16.81	I	75	Yes	65
HIV Neur	42	M	23.31	I	<47	Yes	24
HIV Neur	50	M	20.67	II	452	Yes	12
HIV Neur	61	M	18.58	I	<47	Yes	32
HIV Neur	56	F	19.38	II	521	Yes	4
HIV Neur	47	M	30.06	IV	4299	N-A	N-A
HIV Neur	55	M	30.10	I	148	Yes	65
HIV Neur	40	F	24.84	IV	N-A	Yes	65
HIV/HTLV Neur	40	F	22.83	I	<47	Yes	51
HIV/HTLV Neur	36	M	17.08	I	1051596	Yes	36
HIV/HTLV Neur	35	F	17.10	II	49	Yes	23
HIV/HTLV Neur	39	F	21.80	I	73347	N/A	0
HIV/HTLV Neur	37	F	24.92	I	1407	Yes	61
HIV/HTLV Neur	50	F	17.40	I	114	Yes	63
HIV/HTLV Neur	50	M	25.18	I	<47		12
HIV/HTLV Neur	50	F	33.59	I	N-A	N/A	0

*HIV Neur* HIV infected with neurological symptoms, *HIV*/*HTLV Neur* HIV and HITLV co-infected with neurological symptoms, *BMI* Body mass index, *M*/*F* male/female, *N*/*A* not applicable, *N-A* Not available

### Increased frequency of *CD4*^+^*CD25*^*High*^*T cells in* HIV-1/HTLV-1 co-infected patient*s*

As shown in Fig. 1, higher absolute (median and interquartile range (IQR) = 1076 cells/mm^3^ and 483 cells/mm^3^) and relative counts of CD4 T cells (median and IQR = 37.5 and 11 %) were found in HIV/HTLV Neur patients, when compared with the HIV Neur group (median and IQR = 275 cells/mm^3^ and 152 cells/mm^3^, *p* = 0.0018; median and IQR = 18 and 11 %, *p* = 0.0018, respectively). However, no significant difference was found between HIV/HTLV Neur and control groups (*p* = 0.1877). Moreover, we did not find differences with regards to CD8^+^ T cells, NK cells and B cell counts between the two HIV infected groups.

Lower numbers of CD4^+^CD25^Low^ T lymphocytes were seen in HIV Neur group (median and IQR = 1.8 and 2.1 %) when compared with controls (median and IQR = 4.1 and 2.6 %, *p* = 0.028). However, the difference between these two groups in terms of frequency of CD4^+^CD25^High^ T lymphocytes did not reach a significant level (median and IQR = 0.45 and 0.8 %; median and IQR = 1.2 and 0.4 %, respectively, *p* = 0.062). There was higher expression of both CD4^+^CD25^Low^ and CD4^+^CD25^High^ T cells (median and IQR = 21.1 and 7.6 %; median and IQR = 25.9 and 16.8 %, respectively) in the HIV/HTLV Neur group when compared with the HIV Neur group (*p* = 0.0008 and *p* = 0.0007, respectively) and the control group (*p* = 0.0034 and *p* = 0.0034, respectively).

### *Increased frequency of* CD4^+^CD25^High^Foxp3^+^*Treg cells in HIV*-*1*/*HTLV*-*1 co*-*infected patients*

**Fig. 2:**
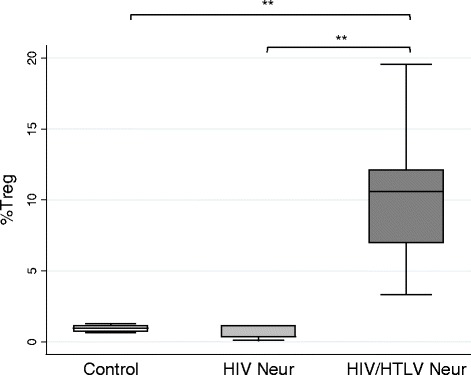
Frequency of Treg cells. Median of Treg cells (CD4^+^CD25^High^Foxp3^+^), expressed as percentage in relation of total number of lymphocytes, are shown for each group. Control (*n* = 4); HIV Neur (*n* = 7); HIV/HTLV Neur (*n* = 6). **p < 0.005

We first showed that HIV/HTLV Neur patients exhibited higher numbers of Treg cells (median and IQR = 10,6 and 6.6 %) when compared with the control (median and IQR = 0.9 and 0.3 %, *p* = 0.0105) and HIV Neur groups (median and IQR = 0.4 and 0.7 %, *p* = 0.0027). To assess the influence of the frequency of absolute CD4^+^ T cells on the above facts, we determined the Treg proportional values among total CD4^+^ T cells (Fig. 2). Co-infected individuals HIV/HTLV Neur had a significantly higher proportion of Treg cells (median and IQR = 0.2 and 0.2, respectively), compared with both control (median and IQR = 0.02 and 0.01, *p* = 0.007) and HIV Neur individuals (median and IQR = 0.03 and 0.02, *p* = 0.014).

### Low levels of Foxp3 and higher levels of CD49d expression in CD4^+^CD25^High^Foxp3^+^ Treg cells from HIV-1/HTLV-1 co-infected patients

**Fig. 3:**
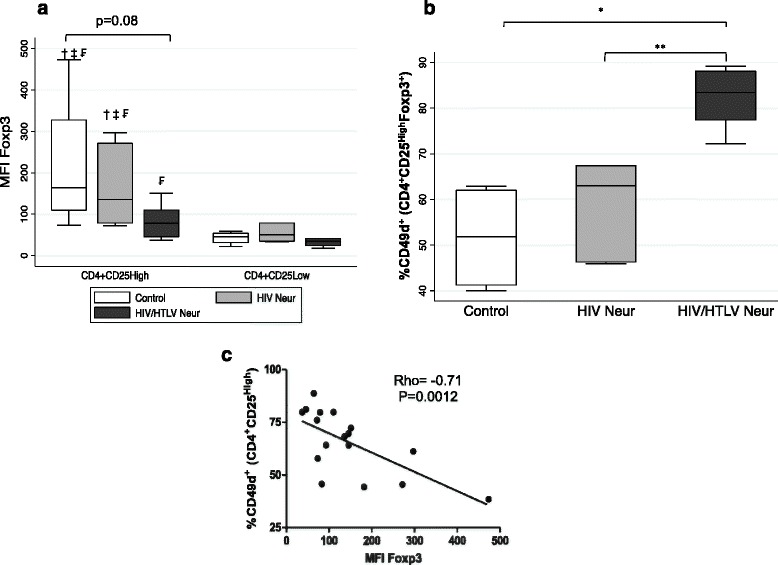
Density of FoxP3 in CD4^+^CD25^High^ cells and their correlation with frequency of CD49d. In (**a**) median fluorescence intensity of CD4^+^CD25^High^ and CD4^+^CD25^Low^ cells from individuals of control (*n* = 4), HIV Neur (*n* = 7) and HIV/HTLV Neur (*n* = 6) groups. †, ‡ and ₣, difference statistically significant in relation to CD4^+^CD25^Low^ cells from control group, HIV Neur and HIV/HTLV Neur, respectively. Note that there is a significant difference in the density of Foxp3 expression between CD4^+^CD25^High^ and CD4^+^CD25^Low^ cells in the same group. In (**b**) frequency of CD49d in Treg cells. *p < 0.05, **p < 0.001. In (**c**) correlation between CD49d and density of FoxP3 expression in CD4^+^CD25^High^ cells, obtained as the median fluorescence intensity (MFI)

We observed a trend towards lower expression of Foxp3 in the HIV/HTLV Neur group when compared with the HIV Neur group (*p* = 0.15) and the control group (*p* = 0.08), although the differences were not statistically significant (Fig. 3a). However, we noticed that in the HIV/HTLV Neur group, the MFI level for Foxp3 within the subset of CD4^+^CD25^High^ cells did not differ from those observed in CD4^+^CD25^Low^ cells (effector cells) of control (*p* = 0.13) and HIV Neur groups (*p* = 0.25).

In addition, in HIV mono-infected individuals presenting neuropathy, there was a higher, but not statistically significant, frequency of the inflammatory marker CD49d in Treg cells (median and IQR = 63 and 21.1 %) compared to the control group (median and IQR = 51,8; 20.7 %, *p* = 0.167). A higher percentage of CD49d^+^ cells bearing the Treg phenotype were indeed detected in the HIV/HTLV Neur group (median and IQR = 83.4 and 10.7 %) compared to controls (*p* = 0.0105) and HIV Neur group (*p* = 0.0027) (Fig. 3b). We also found a higher density of CD49d expression in cells of this group (median and IQR = 319.52 and 97.76) compared to controls (median and IQR = 138.16 and 21.12, *p* = 0.0105). There was an inverse correlation between the frequency of CD49d and the MFI of Foxp3 (rho = −0.71, *p* = 0.0012) (Fig. 3c).

### Positive correlation between activated CD4^+^HLA-DR^+^ cells and cells presenting the Treg phenotype in HIV-1/HTLV-1 co-infected group

**Fig. 4:**
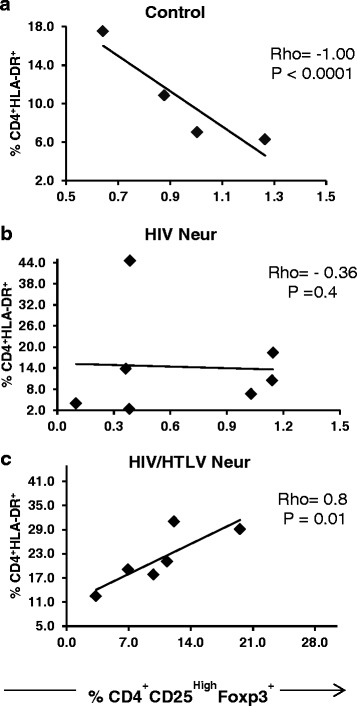
Correlation between the frequency of Treg cells and CD4+ activated lymphocytes. In **a**, **b** and **c**, correlation between the frequency of Treg cells and CD4 activated cells (CD4^+^HLA-DR^+^) in control HIV Neur and HIV/HTLV Neur groups. Only in co-infected group a positive correlation was seen

We found that the percentages of activated CD8^+^HLADR^+^ cells were not different when HIV Neur (median and IQR = 16.3 and 8.7 %) and HIV/HTLV Neur groups (median and IQR = 12.82 and 12.07 %, *p* = 0.17) were compared, and that there was no correlation between this lymphocyte subset and the Treg cells. On the other hand, the relative frequency of CD4^+^HLADR^+^ lymphocytes was higher in the HIV/HTLV Neur group (median and IQR = 20,1 and 12.54 %), when compared with both the control (median and IQR = 7.04 and 4.5 %, *p* = 0.0084) and the mono-infected group (median and IQR = 8.59 and 12.52 %, *p* = 0.0357). The frequency of activated of CD4^+^HLADR^+^ lymphocytes in the HIV/HTLV Neur group was positively correlated with the frequency of cells bearing the Treg phenotype (rho = 0.8; *p* = 0.01) (Fig. 4).

## Discussion

The results presented herein, phenotypically characterizing Tregs in HIV/HTLV co-infected patients, indicate an impairment of Treg cells in co-infected individuals presenting neurological symptoms. We first observed that the frequency of CD4 T cells with high expression of CD25 was eight-fold higher in HIV/HTLV Neur group than in the mono-infected group. Our data are in keeping with a previous report showing increased frequency of CD4^+^CD25^+^ cells in HIV/HTLV co-infected individuals compared to HIV mono-infected and controls [4]. This effect may be mediated by the Tax encoded by HTLV-1, since this protein is a strong activator of host genes that regulate cell growth, including the gene encoding IL-2 and the alpha chain of its receptor, CD25 [15].

Although we did not find a significant change in Treg counts between the control and the HIV Neur group, it has been described that HIV-1 infected patients receiving ART present higher frequencies of Treg cells, possibly associated with increase in thymic production and export of these lymphocytes [16]. Such divergent results may be due to differences among the cohorts, since in our study we enrolled individuals with neurological dysfunction and CD4 T cell counts less than 350 cells/μL. When analyzing data from the co-infected group, we observed significantly higher absolute and relative Treg cell counts. Our results corroborate previous evidence revealing that HTLV-1 infection leads to an increase in the frequency of Treg cells [17]. It is likely that these changes are due to the spontaneous lymphoproliferation mediated by the Tax protein encoded by HTLV-1. We cannot, however, rule out that the higher frequency of these cells in HIV/HTLV Neur group is merely the consequence of relocation or recruitment of Treg cells from other organs to the circulation. Irrespective of the underlying mechanism, this higher proportion of Treg cells in HIV/HTLV-1 co-infection could favor the modulation of antiviral response.

It has been described that the suppressive function of Treg is associated with the density of Foxp3 expression [18–20]. On the other hand, the Foxp3 density differs between effector and regulatory cells [19, 20]. We observed a mild Foxp3 MFI decrease in HIV/HTLV Neur patients as compared to the control group. Yet, these levels did not differ from those observed for the CD4^+^CD25^low^ effector cells in the control and HIV Neur groups. It is thus possible that in the HIV/HTLV Neur group, cells with the Treg phenotype have impaired function. Reports of Tregs with lower levels of Foxp3 and defective function have been described in HTLV-1 mono-infected patients with HAM/TSP [11, 12]. It has also been shown that reduced levels of Foxp3 in HAM/TSP were correlated with high proviral load and high expression of the Tax-1 protein of HTLV-1 [11].

Under physiologic conditions, higher levels of Tregs will proportionally decrease the frequency of activated T cells. A study conducted in pregnant women found an inverse correlation between Tregs and activated CD4^+^HLA-DR^+^CD38^+^ cells in HIV uninfected but not in HIV infected women [21]. We also found an inverse correlation between the frequencies of activated CD4^+^HLA-DR^+^ cells and Tregs in the control group. Similar to a previous report [4], we found an elevated frequency of CD4^+^HLA-DR^+^ cells in HIV/HTLV Neur patients, which positively correlated with frequencies of Treg cells in the co-infected group, but not in mono-infected individuals. A study conducted in ART naïve HIV infected patients revealed a positive correlation between the frequency of Tregs and CD4^+^HLA-DR^+^CD38^+^ cells in fast progressors but not in slow progressors [9]. Taken together, our results suggest that in HIV infection the rate of CD4 T cell activation is not balanced by Tregs. This unbalance is more evident when the HTLV co-infection is present. Our findings corroborate the notion that the presence of HTLV may favor fast progression to AIDS [4].

Cells with the Treg phenotype expressing the α4 integrin chain (CD49d) of VLA-4 and LPAM-1 may function as pro-inflammatory effector cells that secrete IFN-γ or IL-17 [5]. The removal of such lymphocytes from the pool of regulatory T cells was described as being associated with the induction of a strong suppressive activity [5]. In our study, we found that HIV/HTLV Neur patients presented not only higher frequency of Treg cells bearing the CD4^+^CD25^High^Foxp3^+^CD49d^+^ phenotype, but also showed higher CD49d density. Although we have not evaluated the secretion of inflammatory cytokines by these cells, our data suggest that co-infection by HTLV-1 is associated with higher frequency of CD4^+^CD25^High^Foxp3^+^, broadly defined as potentially inflammatory Treg cells. However further experiments to assess the functionality of these CD4^+^CD25^High^Foxp3^+^ by expression of inflammatory cytokines as IFN-γ and IL-17 need to be conducted to better understand the role of these cells with Treg phenotype. From a standpoint of migratory capacity, the high density of α4 integrin suggests that these cells may migrate efficiently to inflamed CNS by interaction of VLA-4/VCAM and/or to the gut, the primary site of HIV replication, using a mechanism dependent of LPAM-1/MAdCAM interaction.

Infection with HTLV-1 *per se*, enhances expression of the integrin VLA-4 in lymphocytes [22, 23]. Thus, the higher CD49d levels indicate that these cells are preferentially infected with HTLV-1 [24]. CD4^+^CD25^High^Foxp3^+^ cells with potential to migrate to CNS may also be HIV reservoirs [25] and release HIV-1 neurotoxic proteins in the CNS. It has been demonstrated that the HIV-1 Nef protein released into the extracellular environment induces neuron death [25, 26]. Accordingly, it is plausible to conceive that co-infection may act as a co-factor for inducing CNS pathology mediated by either HTLV-1 or HIV-1.

## Conclusions

In summary, the study reported herein indicates a potential dysfunction of Treg cells in patients co-infected by HIV-1 and HTLV-1 presenting neuropathy. Such dysfunction may be related to the inability to control the activation and proliferation of effector lymphocytes, and to changes in the mechanisms that drive Treg traffic. In these patients, it is conceivable that the Foxp3 expression in Tregs is transient, not conveying, therefore, regulatory properties. Rather, Tregs in these patients have the ability to effectively migrate to peripheral tissues, including the gut and the CNS, where they may participate in tissue damage. Yet, we should consider these hypotheses with caution due to limitations of the study, namely the low number of individuals in each group, the absence of HTLV mono-infected group, the use of HLA-DR as the only phenotypic marker of T cell activation and the absence of functional assays to assess the role of these cells with Treg phenotype.

## Abbreviations

AIDS: Acquired Immunodeficiency Syndrome
ART: Antiretroviral therapy
CNS: Central nervous system
CNBS: Mozambican National Committee on Bioethics in Health (Comité Nacional de Biotética em Saúde de Moçambique)
Foxp3: Transcription factor *forkhead box P3*
HAM/TSP: HTLV-1 associated myelopathy/ tropical spastic paraparesis
HIV-1: Human Immunodeficiency Virus type 1
HTLV-1: Human T-lymphotropic Virus type 1
HIV Neur: HIV-infected with neurological symptoms
HIV/HTLV Neur: HIV/HTLV co-infected with neurological symptoms
PBMC: Peripheral blood mononuclear cells
Tregs: Regulatory CD4^+^ T cells
